# Diagnosis and Pathogenesis of Nairobi Sheep Disease Orthonairovirus Infections in Sheep and Cattle

**DOI:** 10.3390/v13071250

**Published:** 2021-06-27

**Authors:** Julia Hartlaub, Benjamin Gutjahr, Christine Fast, Ali Mirazimi, Markus Keller, Martin H. Groschup

**Affiliations:** 1 Institute of Novel and Emerging Infectious Diseases, Friedrich-Loeffler-Institut, 17489 Greifswald–Insel Riems, Germany; julia.hartlaub@fli.de (J.H.); benjamin.gutjahr@fli.de (B.G.); christine.fast@fli.de (C.F.); markus.keller@fli.de (M.K.); 2 Department of Medicine, Karolinska Institutet, SE-17177 Stockholm, Sweden; ali.mirazimi@ki.se; 3 National Veterinary Institute, SE-75189 Uppsala, Sweden

**Keywords:** Nairobi sheep disease orthonairovirus, NSDV, Ganjam orthonairovirus, Crimean-Congo hemorrhagic fever orthonairovirus, CCHFV, pathogenesis, ruminants, serology

## Abstract

Nairobi sheep disease orthonairovirus (NSDV) is a zoonotic tick-borne arbovirus, which causes severe gastroenteritis in small ruminants. To date, the virus is prevalent in East Africa and Asia. However, due to climate change, including the spread of transmitting tick vectors and increased animal movements, it is likely that the distribution range of NSDV is enlarging. In this project, sheep and cattle (hitherto classified as resistant to NSDV) were experimentally infected with NSDV for a comparative study of the species-specific pathogenesis. For this purpose, several new diagnostic assays (RT-qPCR, ELISA, iIFA, mVNT, PRNT) were developed, which will also be useful for future epidemiological investigations. All challenged sheep (three different doses groups) developed characteristic clinical signs, transient viremia and virus shedding—almost independent on the applied virus dose. Half of the sheep had to be euthanized due to severe clinical signs, including hemorrhagic diarrhea. In contrast, the course of infection in cattle was only subclinical. However, all ruminants showed seroconversion—implying that, indeed, both species are susceptible for NSDV. Hence, not only sheep but also cattle sera can be included in serological monitoring programs for the surveillance of NSDV occurrence and spread in the future.

## 1. Introduction

Nairobi sheep disease orthonairovirus (NSDV) is the eponymous representative of the family *Nairoviridae* within the order *Bunyavirales* [1]. Causing severe gastroenteritis in small ruminants with case fatalities of 30–70% in susceptible populations, NSDV is part of the OIE list for notifiable animal diseases [2]. Due to its zoonotic impact, NSDV is classified as a biosafety level (BSL) 3 agent, but reports of human infections resulting in febrile illnesses are scarce [3,4,5]. In contrast, the distantly related Crimean-Congo hemorrhagic fever orthonairovirus (CCHFV, BSL 4) can induce fatal disease in humans. Leading both to hemorrhagic fevers in small ruminants and humans, NSDV has already been proposed as a model for human CCHFV infections [6,7].

The single-stranded RNA genome of NSDV is divided into three segments. The nucleocapsid protein (N) is encoded by the small (S) segment, the glycoprotein precursor (GPC) by the medium (M) segment, and the RNA-dependent RNA polymerase by the large (L) segment, respectively [8,9].

In 1910, high mortality rates were observed in sheep, which were brought to Nairobi livestock markets from the Masai region of Kenya. The causative agent—NSDV—was isolated out of several affected sheep and *Rhipicephalus appendiculatus* ticks were identified as the main vectors for transmitting this disease. As no direct transmission between infected sheep could be demonstrated, the prevalence of NSDV is thought to be directly linked to the natural distribution range of its tick vectors [10].

NSDV has caused several epizootics in East Africa (e.g., Kenya, Uganda, Somalia, Ethiopia, Tanzania and Rwanda [10,11,12,13,14,15]), but no large outbreaks were reported in the last few decades. Serological evidence for NSDV occurrence also exists in Southern Africa (e.g., South Africa, Mozambique), but these observations may be due to serological cross-reactions as no NSD-like disease has been reported there yet [16].

Concerning the epidemiological situation in endemic regions, it is hypothesized that young sheep are exposed to the virus during the period in which they are still partially protected by maternal antibodies. Hence, they do not succumb to the disease and develop their own adaptive immune response, which protects them during their whole lifetime [17]. It is assumed that virtually all sheep have NSDV antibodies in these endemic areas. This stable state can be interrupted, if susceptible (naïve) animals are introduced to these regions or if the distribution range of the transmitting tick vector is enlarging. Both options are likely to occur, when climate change in combination with vector spread, optimization of land use as an answer for the globally increased demand for meat, and the importation of foreign animals for breeding improvements are taken in consideration [18].

The most momentous finding in this century was that NSDV is also prevalent in Asia (India [19], Sri Lanka [20]). In 1954, Ganjam virus (GANV) was isolated out a pool of *Haemaphysalis intermedia* ticks in Orissa, India [21]. The serological relationship between NSDV and GANV was demonstrated [22] and sequence analyses proved that they were indeed identical [23,24]. Moreover, viral NSDV RNA was also detected in *Haemaphysalis longicornis* ticks in different regions in China; implying an even larger distribution range of NSDV in Asia [25,26].

Recently, the potential spread and emergence of NSDV was modulated with an ecological niche model and countries at risk (e.g., Ethiopia, Malawi, Zimbabwe, Southeastern China, Taiwan, Vietnam) were suggested, where NSDV might (already or in the future) occur and cause outbreaks [18]. To increase the preparedness for a potential emergence of this neglected arbovirus, further research concerning the pathogenesis, transmission and diagnosis of NSDV is essentially needed. For the determination of the actual distribution of NSDV, the development of new sensitive and specific diagnostics is obligatory. In former studies on outbreaks mainly suckling mouse or sheep inoculations were conducted to obtain virus isolates. Complement fixation (CF), hemagglutination inhibition (HI) or in-vivo mouse neutralization assays were used for serological analyses [27,28,29,30]. However, no standardized modern diagnostic test is currently available for the detection of NSDV RNA (RT-qPCR) or NSDV specific antibodies.

For the development and evaluation of such diagnostic assays, defined virus- and antibody-positive reference sera are essential. In the presented report, sheep and cattle were experimentally infected with NSDV, in order to study the species-specific pathogeneses and obtain these reference samples. One study was recently published and described the experimental infection of sheep with two different strains of NSDV, but no information considering the immune responses were included, as all sheep were necropsied up to 11 days post infection [6]. In consequence, the main focus of our study was put on the serological part. However, we also included some new aspects concerning the acute phase of infection (swab sampling to evaluate potential shedding of the virus, application of different infection doses, RT-qPCR analyses of organ samples). Moreover, we decided to include cattle in our study which were considered being refractory to NSDV infection, as Montgomery stated that infected cows did not develop clinical signs [10]. However, subclinical infections might still occur and play a role in the natural infection cycle. So far, no vertebrate host is known as a reservoir or subclinical host for NSDV, although experimental infections of African grass rats (*Arvicanthis abyssinicus nairobiae*) induced a short viremia in this species, which was sufficient to infect ticks [14,31]. Cattle are frequently infested with the same tick species such as small ruminants, and are susceptible to the closely related viruses CCHFV [32] and Dugbe orthonairovirus (DUGV) [33]. They do not show any clinical signs following these infections—hence, this might probably be also true for NSDV.

## 2. Materials and Methods

### 2.1. Virus and Cells

The prototype strain of Ganjam orthonairovirus (IG619, GenBank accession number: KU925466, KU925465, KU925464) was utilized for all in vitro and in vivo studies involved in this project (kindly provided from the World Reference Center for Emerging Viruses and Arboviruses, University of Texas Medical Branch, Galveston, USA). The lyophilized virus stock (6th suckling mouse brain passage) was grown on human adrenocortical carcinoma (SW13) cells. These were a kind gift from Ali Mirazimi, National Veterinary Institute, Sweden. Cells were cultivated with L-15 Medium (Sigma Aldrich, Darmstadt, Germany), supplemented with 5% fetal calf serum (FCS). Prior to the animal trials, the stock was passaged once again on SW13 cells. Growth kinetics revealed maximum viral titers 36 h post infection (up to 10^7.9^ TCID_50_/mL). Vero E6 cells (Collection of Cell Lines in Veterinary Medicine, Friedrich-Loeffler-Institut, Germany) were utilized for the indirect immunofluorescence assays (cultivation with Modified Eagles Medium (MEM, Collection of Cell Lines in Veterinary Medicine, Friedrich-Loeffler-Institut, Germany) supplemented with 10% FCS)).

### 2.2. Quantification

The methods for the quantification of viral titers were previously described [34]. Briefly, two different assays were performed utilizing SW13 cell monolayers. In a 96-well format, end-point titrations were performed, and the cytopathic effect (CPE) was evaluated 7 days post infection (dpi) for the calculation of TCID_50_ titers (50% Tissue Culture Infective Dose). In a 6-well format, plaque assays were performed to obtain titers expressed as plaque forming units (pfu). Fixation and staining of the plaque assay were performed 5 dpi. Both assays lead to comparable titers for the same virus stock (10^7.2^ TCID_50_/mL vs. 10^7.1^ pfu/mL).

All RT-qPCR positive samples, which were collected during the animal trials, were subsequently titrated for the determination of TCID_50_ titers in order to investigate, whether infectious virus particles were present in the serum, swab and organ samples. Therefore, each sample was titrated in quadruplicate. To rule out non-specific cytotoxic CPE (not related to actual virus growth), plates were additionally inspected at two- or three-days post infection.

### 2.3. Quantitative Reverse Transcriptase Polymerase Chain Reaction (RT-qPCR)

To determine the load of viral genome equivalents in samples suspected to be positive for NSDV, a TaqMan probe, compatible with primers of a published SYBR-Green-based RT-qPCR [6] targeting the 5′ part of the coding region on the S segment, was generated. The primers and probes that were used are listed in Table 1.

For the quantitative detection of the NSDV genome by RT-qPCR, the NSDV specific primer and probe are used (Table 1). As an internal extraction control, MS2 bacteriophage RNA was added to the samples before RNA isolation. For the detection of MS2 RNA, a specific primer/probe mix was used in a duplex approach [35]. The reaction mix was set up using the QuantiTect Probe RT-PCR Kit (Qiagen, Hilden, Germany) in a total reaction volume of 25 μL according to manufacturers’ instructions. Then, 5 µL template RNA was added to the reaction mix, comprising 12.5 µL 2x reaction master mix, 0.25 µL RT-Mix, 2 pmol of each NSDV primer, 0.5 pmol of NSDV probe, 10 pmol of each MS2 primer, 4 pmol of MS2 probe and water to fill up to a volume of 20 µL. For quantification, a synthetic RNA calibrator generated by in vitro transcription from the corresponding DNA-sequence, which contains an additional T7 promotor sequence, was used. This synthetic calibrator comprises the target region of the RT-qPCR and also serves as a positive control on each RT-qPCR plate.

Cycle conditions on a CFX96 Real-Time PCR Detection System (Bio-Rad Laboratories, Hercules, CA, USA) were as follows: 50 °C for 30 min (reverse transcription), 95 °C for 15 min (reverse transcriptase inactivation/Taq polymerase activation), followed by 48 cycles at 95 °C for 15 s (denaturation), 63 °C for 30 s (annealing) and 72 °C for 30 s (elongation). After each elongation step fluorescence data were collected and subsequently analyzed with the CFX Manager software (Bio-Rad Laboratories, Hercules, CA, USA).

### 2.4. Recombinant Expression of NSDV N Protein

For the recombinant expression of NSDV N protein, the nucleic acid sequence located on the S segment of NSDV reference strain IG619 (Genbank accession number KU925466) was codon optimized for expression in E. coli. For subcloning into the bacterial expression vector pET19b, recognition sites for NdeI and BamHI were added to 5′ and 3′ of the coding sequence, respectively. The resulting synthetic plasmid, comprising restriction sites and coding sequence, were generated by Eurofins Genomics (Ebersberg, Germany). After subcloning into pET19, sequence identity and the correct insertion of the N protein coding sequence was verified by Sanger sequencing.

Plasmid pET19b-NSDV N was transformed into E. coli K12 strain BL21(DE3) and the expression of the N protein was induced by the addition of Isopropyl-β-d-thiogalactopyranosid (IPTG, MP Biomedicals, Irvine, CA, USA) during the logarithmic growth phase of the bacteria. Growing bacteria were harvested after 4 h of expression and, after sedimentation, bacteria were lysed and NSDV N protein was isolated and purified using the N-terminal His-Tag binding to Ni-NTA Sepharose, utilizing the customary Expressionist protocol (Qiagen, Hilden, Germany).

### 2.5. Serology

#### 2.5.1. Development of an Indirect IgG-ELISA Based on Recombinant Antigen

Bovine protocol: The recombinant NSDV N protein was diluted in phosphate-buffered saline (PBS) containing 0.5% BSA (Albumin fraction V, Merck, Darmstadt) to a final concentration of 2 µg/mL. For every serum sample, two wells of a Greiner F plate (greiner Bio-One GmbH, Frickenhausen, Germany) were coated with the NSDV antigen and two with the antigen dilution buffer only. In total, 100 µL per well were coated overnight at 4 °C. After a washing step (three times 200 µL per well, washing solution: PBS, 0.1% Tween20), plates were blocked with IDVet blocking solution (IDVet, Grables, France) for 1 h at room temperature. Plates were then pushed on cellulose for drying. Sera were diluted 1/20 in IDVet buffer No. 3 and 100 µL/well were added. On every plate, one positive control (NSDV-IMMU-calf-1, pre-diluted 1/20 in negative cattle serum) and negative control serum were included. Incubation of the plates was performed at 37 °C for 1 h. Afterwards, plates were washed again three times with the washing solution, before 100 µL of the 1/15,000 in IDVet buffer No. 11 diluted secondary antibody (Peroxidase-conjugated AffiniPure Goat Anti-Bovine IgG, Jackson ImmunoResearch, Cambridgeshire, UK) were added to each well and plates were incubated for 1 h at 37 °C. After rinsing the plates three times with the washing solution, 100 µL of the substrate (1-Step^TM^ Ultra TMB-ELISA, Thermo Scientific, Braunschweig) were added to each well and incubated approx. 15 min in the dark. Reaction was stopped with 100 µL/well 1M H_2_SO_4_ and OD values were measured at 450 nm. For every sample, the corrected OD values were determined (mean OD_450_ with antigen–mean OD_450_ without antigen) and the percentage of this OD value in comparison to the positive control was calculated.

For the ovine N protein-based ELISA, the bovine ELISA protocol was modified in several aspects: Recombinant N protein was diluted in PBS containing 0.5% albumin from sheep (Sigma Aldrich, Darmstadt, Germany). The anti-species antibody (Peroxidase-conjugated AffiniPure Donkey Anti-Sheep IgG, Jackson ImmunoResearch, Cambridgeshire, UK) was diluted 1/3000. As a positive control, and as the basis for the calculation of corrected OD values, the serum of an immunized sheep (NSDV-IMMU-sheep-1) was utilized. In contrast to the bovine protocol, this serum was diluted in the same manner as the test sera. Plates were incubated for approx. 20 min with the ELISA substrate.

#### 2.5.2. Indirect Immunofluorescence Assay (iIFA)

The indirect immunofluorescence assay (iIFA) was performed as already described [34]. Vero E6 cells were infected with NSDV (IG619) and fixed with methanol/acetone. Test sera were diluted 1/50 and a species-specific secondary antibody was added afterwards. To exclude non-specific reactions, sera were also tested on non-infected cells.

#### 2.5.3. Plaque Reduction Neutralization Test (PRNT)

The protocol for the Plaque Reduction Neutralization Test (PRNT) was already published [34]. Briefly, serial serum dilutions were incubated with approx. 50 pfu NSDV, before the serum/virus mixture was transferred to prepared SW13 cell monolayers in a 6-well format. After an incubation period, the inocula were removed and an overlay was added to each well. Plates were fixed after 5 days, and a serum dilution was qualified positive if 80% of plaques (PRNT_80_) were reduced compared to the control plate.

#### 2.5.4. Micro-Virus Neutralization Test (mVNT)

The mVNT was performed as already described [35]. Briefly, serial serum dilutions were incubated with approx. 100 TCID_50_ NSDV, before the serum/virus mixture was transferred to the prepared SW13 cell monolayers. Plates were fixed after 7 days and ND_50_ titers (50% Neutralizing Dose) were calculated according to Behrens-Kaerber.

### 2.6. Immunizations

Serving as positive controls in different assays, one calf (NSDV-IMMU-calf-1) and one sheep (NSDV-IMMU-sheep-1) were immunized with formalin-inactivated NSDV (cattle: 3 boosts with 10^7.5^ TCID_50_, sheep: 3 boosts with 10^8.1^ TCID_50_). The boosts contained the inactivated virus and equivalent amounts of adjuvant (GERBU Adjuvant P, GERBU Biotechnik GmbH, Heidelberg, Germany). The inocula were subcutaneously injected at four different locations (2.5 mL per injection site).

### 2.7. Animal Trials

All animal trials were conducted under BSL 3 containment conditions. Twelve sheep (German mutton, 6 female/6 male, age 4 months, obtained from Friedrich-Loeffler-Institut, Mariensee) were subcutaneously inoculated into the lateral chest wall (injected volume: 3 mL). Three different challenge doses were applied: four sheep were infected with 10 TCID_50_ (internal animal codes: N1–4), four sheep with 10^3^ TCID_50_ (M1–4) and another four sheep with 10^5^ TCID_50_ (P1–4) NSDV. These different infection doses were chosen to mirror different infection patterns. It was expected that the animals of the higher dosage groups (P, M) would develop severe signs (and eventually might have to be euthanized due to animal welfare reasons), whereas animals from the low dosage group should all have survived and developed NSDV-specific antibodies for our serological investigations. Two sheep (O1, O2) were kept as mock controls in a separate stable and were only inoculated with cell culture medium.

Four calves (Holstein Friesian, 2 female/2 male, age 5 months, obtained from commercial supplier RinderAllianz GmbH and from Friedrich-Loeffler-Institut, Mariensee, internal animal code: Q5, Q6, R5, R6) were subcutaneously inoculated with 10^6^ TCID_50_ NSDV. As we did not expect the calves to be highly susceptible to NSDV, we decided to apply a higher challenge dose and inoculated them with the similar virus titers, as previously described [34,35]. The corresponding control animal (calf S2) was only inoculated with cell culture medium and kept together with the control sheep.

During the observation period, rectal body temperatures of all animals were measured daily and clinical scores (considering the general behavior and condition, as well as injuries, breathing rates, food uptake and diarrhea) were determined. One physical examination every morning was at least accompanied by one or two camera-assisted observations during the day. Animals that developed severe clinical signs were intravenously euthanized with pentobarbital (Release^®^, WDT, Garbsen, Germany) and necropsied. Animals that survived the acute phase of infection were kept until 26–28 days post infection.

Several samples (serum, EDTA blood, nasal and rectal swabs) were collected prior to infection and at defined time points during the experiment. In the first week, samples were collected every day, alternating half of the animals; the surviving animals were again divided in two groups and each group was alternately sampled only every second day during the remaining observation period.

During the necropsies, multiple tissue samples were collected for RT-qPCR analyses as well as for further pathohistological investigations: alimentary tract: rumen, abomasum, duodenum, jejunum *, ileum, caecum, colon *, rectum *, liver *, gall bladder; respiratory tract: conchae, lung *; lymphatic tissue: Lnn. retropharyngeales med *, Lnn. iliaci med *, tonsils *, Ln. cervicalis cran *, Lnn. mesenteriales, Lnn. hepatici, spleen *; nervous system: brain *, coeliac ganglion *; others: heart *, kidney *, injection site (lateral chest wall) *, conjunctiva. For the bovines, a reduced organ panel was collected, as no gross lesions were remarkable (marked with an *).

A tissue homogenizer (geneye UPHO, nbs scientific, Weinheim, Germany) was utilized in order to obtain organ homogenates. Total RNA from all collected samples was extracted (King Fisher 96 Flex purification system, Thermo Scientific, Braunschweig, Germany in combination with the NucleoMag Vet Kit (Macherey-Nagel, Düren, Germany)). To assure the successful RNA extraction, an MS2 bacteriophage was also added. This internal extraction control is required to avoid false negative results in the following NSDV-RT-qPCR due to inaccurate RNA extraction.

## 3. Results

### 3.1. Clinical Signs and Gross Pathology

Twelve sheep, as well as four calves, were experimentally infected with NSDV. The sheep were divided in groups of four individuals that received the same virus dose (10^1^, 10^3^, 10^5^ TCID_50_ NSDV). During the observation period (4 weeks) all animals were monitored daily and several samples (whole blood, serum, swabs) were collected at defined time points post infection.

**Figure 1:**
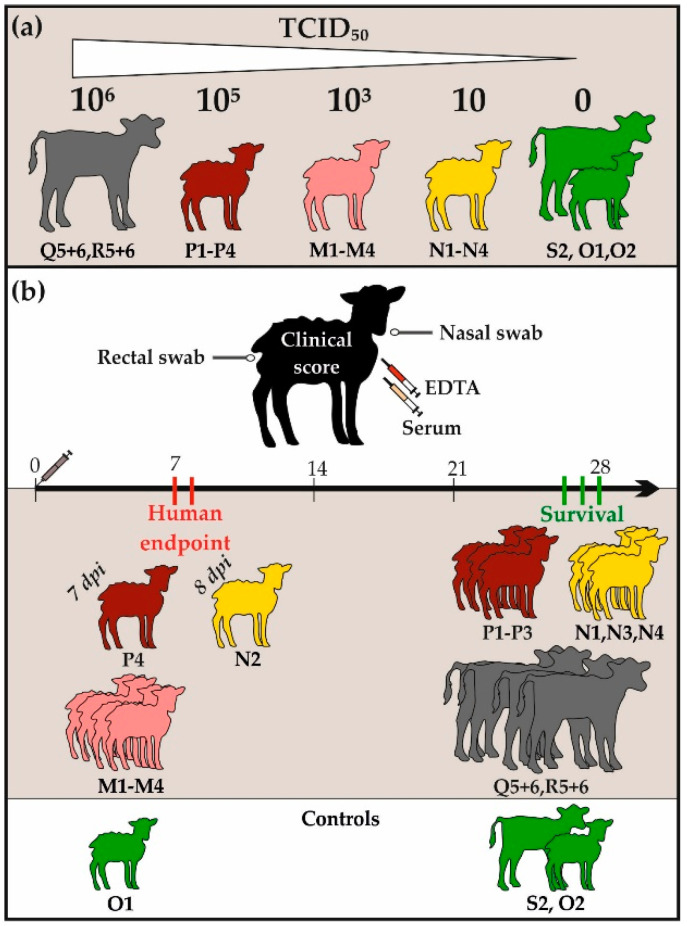
Experimental set-up and clinical outcomes: All animals involved in this study are visualized alongside with the corresponding infection doses in section (**a**). In section (**b**), the collected samples are presented together with the clinical outcomes of all infected animals. Whereas half of the sheep had to be euthanized due to human endpoints, all calves and the other six sheep survived the NSDV challenge.

Due to severe clinical signs, all sheep of the middle dosage group and one animal each from the high and low dosage group had to be euthanized 7 (M1–4, P4) or 8 (N2) days post infection. The other sheep, as well as all infected calves, survived until the end of the experiment (26–28 dpi). One control sheep (O1) was necropsied at 7 dpi as a negative control for pathological investigations (experimental set-up visualized in Figure 1).

**Figure 2:**
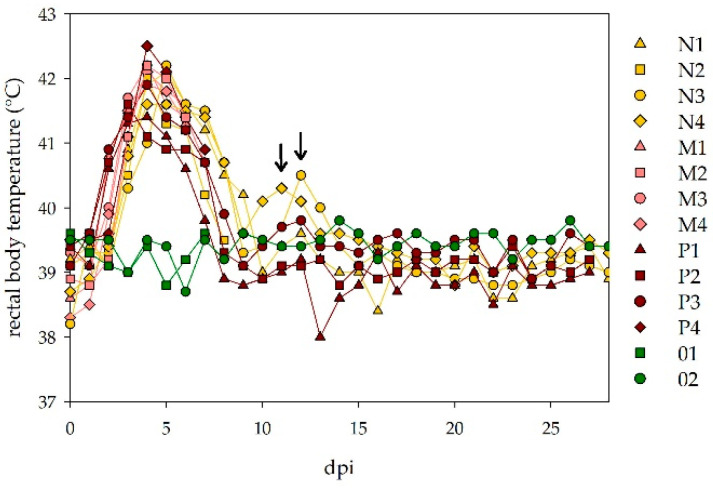
Rectal body temperatures of challenged sheep: all twelve infected sheep developed high fever following the NSDV inoculation, almost independent of the applied virus dosage. Two animals of the low dosage group (N3, N4) showed a second increase in their rectal body temperature (marked with arrows). The control animals did not develop an increase in their rectal body temperatures.

All twelve sheep developed high fever after an incubation period of 2–3 days. Peak rectal body temperatures were reached 3–5 dpi with maximum temperatures of 42.5 °C. The fever period lasted for 5–7 days. For two sheep of the low dosage group (N3, N4) a second increase in the rectal body temperature was seen with another fever peak 11- or 12-days post infection (Figure 2).

Starting with the decline in the rectal body temperature, clinical signs were recognized with a varying degree for the individual animals. All sheep developed diarrhea, which began dark and watery and later—in some animals—contained blood and mucus membrane constituents. A strong loss of appetite was observed and the animals were in a poor body condition after starving for three days. Additionally, conjunctivitis and nasal discharge was seen in most of the sheep. Due to severe clinical signs, six sheep were euthanized at 7 dpi, when their fever was still present, and the animals had become very weak following the diarrhea, dehydration and anorexia. One sheep (N2) had to be euthanized at 8 dpi when it showed severe depression, disorientation and became recumbent. The remaining six sheep started to feed again and were gradually in a better condition. From day 11 onwards, no further clinical signs were seen and the animals fully recovered until the end of the experiment.

As expected, at gross examination distinct differences between the sheep euthanized during the clinical phase of disease (6/12) and the sheep killed at the end of experiment (6/12) were obvious. All animals killed at 7 or 8 dpi showed more and widespread lesions. This includes, besides a weak condition score (6/6) and external signs of diarrhea, a distinct depletion of Peyer’s patches (3/6), clear signs of an enteritis (2/6), acute hemorrhages in the gut (3/6) as well as mild subepicardial hemorrhages (4/6). Two (2/6) sheep also showed a pale and fragile liver. In contrast, sheep which were necropsied at the end of the experiment were mostly in a good body condition (4/6), but two (2/6) animals revealed a weak condition score. In the gut of some animals (3/6) a distinct increase in solitary follicles was found and one animal showed a mild chronic interstitial nephritis. Control sheep were in a good body condition and showed no lesions typical for viral diseases.

None of the infected calves developed clinical signs or a change in the rectal body temperature (data not shown). No gross lesions related to viral disease were seen during the necropsies.

### 3.2. Viral Genome Detection and Virus Titrations

#### 3.2.1. Viremia

**Figure 3:**
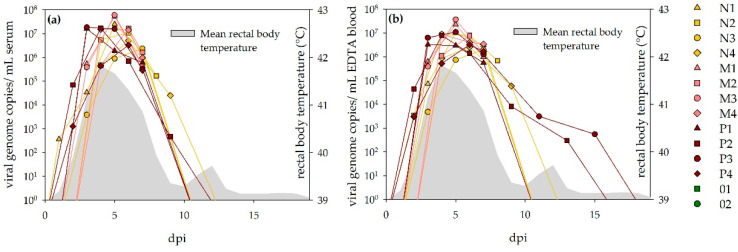
Viremia detected via RT-qPCR in sheep: Results for serum (**a**) and EDTA (**b**) are depicted, respectively. Both curves are comparable between the different dosage groups and sample sources. For two animals (P3, P4), virus was detected in EDTA blood for significantly longer than in the corresponding serum samples. The viremic state coincided well with the fever period (grey pattern).

All sheep developed transient viremia following the NSDV infection. Curves displaying the corresponding RT-qPCR results are depicted for serum and whole blood (Figure 3). Viremia started 1–4 dpi and reached its maximum 3–6 dpi. Duration of the viremic state was between 5 and 8 (serum) or 13 (EDTA) days, respectively.

All positive RT-qPCR serum samples were titrated. Peak values were reached at 3–6 dpi, with a maximum titer of 10^5^ TCID_50_/mL serum for sheep P1, 3 dpi (Table 2).

The calves did not develop detectable viremia via RT-qPCR.

#### 3.2.2. Virus Shedding

**Figure 4:**
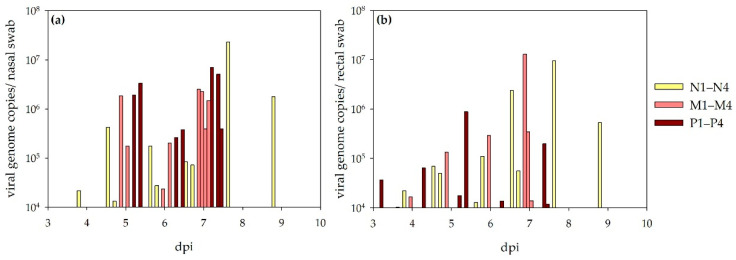
Virus shedding detected via RT-qPCR in sheep: results for nasal (**a**) and rectal swabs (**b**) are depicted. Viral RNA was detected as being steadier in nasal swab samples compared to rectal swab samples.

During the observation period, nasal and rectal swab samples were collected regularly. RT-qPCR results are depicted on Figure 4. All sheep shed the virus via the respiratory and alimentary tract. The shedding started between 3 and 6 (nasal swabs) or 5 (rectal swabs) days and reached its maximum between 4 (rectal swab) or 5 (nasal swab) to 9 days. All samples were titrated, but no virus specific CPE became obvious in any of the collected swab samples.

The bovines did not shed the virus, as all RT-qPCR swab samples were tested negative.

#### 3.2.3. NSDV Organ Distribution

**Figure 5:**
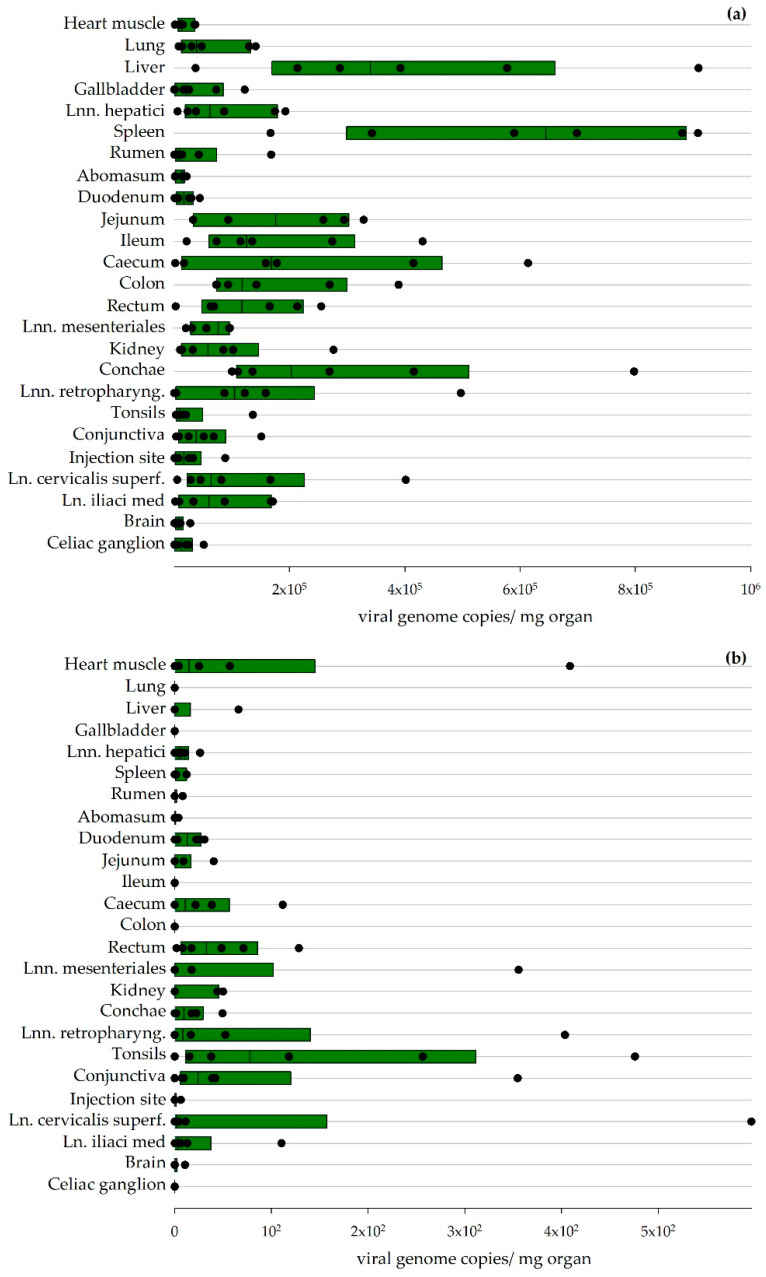
Viral organ loads detected via RT-qPCR in sheep: Results for sheep euthanized 7/8 dpi (**a**) and 27/28 dpi (**b**) are depicted. The boundaries of the boxes indicate the 25th and 75th percentiles, and the line within the box marks the median (calculation performed by Sigmaplot).

In total, 25 tissue samples were collected from each sheep during the necropsies. Viral genome copies were detected in every tissue sample for the sheep necropsied 7 or 8 dpi. The liver and spleen yielded the highest viral loads, but virus could also be detected in the intestines and lymphatic tissues and all other tissue samples (Figure 5a).

All RT-qPCR positive samples were titrated, and the viral titers are presented in Table 3. Virus-specific CPE was seen for all six sheep of the acute phase of infection for the conchae and for five sheep in the conjunctiva and spleen, respectively, but viral titers could also be determined for even more organs in an animal-specific pattern.

RT-qPCR analysis of the various organs collected from sheep 27/28 dpi revealed low virus loads mainly in lymphatic tissues (Figure 5b). The titration of these organs did not lead to a positive result.

### 3.3. Serology

#### 3.3.1. Indirect IgG ELISA Based on Recombinant Protein

**Figure 6:**
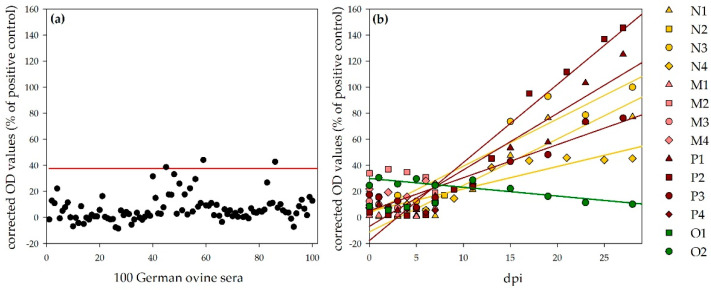
NSDV ELISA results for ovines: Panel (**a**) shows the results for 100 German ovine reference sera for the determination of the negative cut-off (illustrated by the red line; specificity, 97%). Panel (**b**) shows the results for the challenged sheep. Whereas the OD values for the control sheep (O2) stayed on the same level, linear regressions (calculation performed by Sigmaplot) show a steady increase in OD values for the infected sheep, which survived until the end of the observation period.

**Sheep:** For the determination of a negative cut-off value for the ovine NSDV ELISA, 100 sera from German sheep were tested with this ELISA protocol. The cut-off value was calculated as follows: mean + 3 × standard deviation = 37.5% (specificity: 97%, Figure 6a). All sera, which were collected during the animal trials, were further analyzed with this ELISA. Corrected OD values (percentage of the positive control, NSDV-IMMU-sheep-1) rank from 0.7% (N1) to 33.8% (M2) for sera prior to infection, which is within the range of the other 100 negative reference sera. Linear regressions of corrected OD values show a steady increase for all infected sheep, which survived until the end of the experiment (27/28 dpi: N1: 77.1%; N3: 99.9%; N4: 45.2%; P1:125.0%; P2: 145.5%; P3: 76.2%), whereas the tested control sheep O2 was negative during the whole period (e.g., O2, 28 dpi; 10.1%; Figure 6b). The time points, when the negative cut-off value was passed by the individual animals, are listed in Table 4. The sheep that were necropsied 7 or 8 dpi did not develop N protein-specific antibodies, detectable with the newly developed ELISA (7/8 dpi: N2: 17.0%; M1: 7.1%, M2: 19.0%; M3: 10.9%; M4: 25.5%; P4: 5.4%).

**Figure 7:**
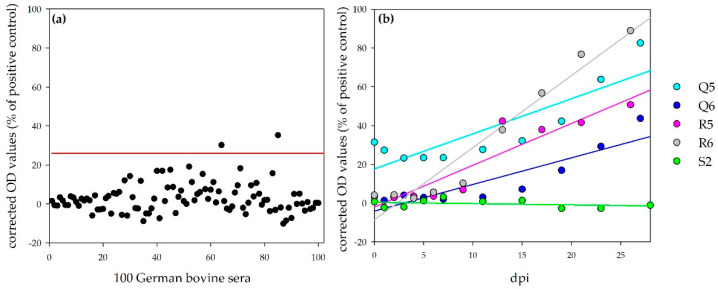
NSDV ELISA results for bovines: Panel (**a**) shows the results for 100 German bovine reference sera for the determination of the negative cut-off (illustrated by the red line; specificity, 98%). Panel (**b**) shows the results for the challenged calves. Whereas the OD values for the control calf (S2) stayed on the same level, linear regressions (calculation performed by Sigmaplot) show a steady increase in OD values for the infected calves.

**Calves:** In total, 100 sera from German cattle were tested with the NSDV ELISA for the determination of the negative ELISA cut-off (mean + 3 × standard deviation = 26.0%, Specificity: 98%, Figure 7a). Linear regression of the results for the sera of the four infected calves show a steady increase during the observation period (Figure 7b). One calf was already tested as weakly positive prior to infection (Q5, 0 dpi, 31.5%), but reacted comparable to the other calves with an increase in OD values afterwards (26/27 dpi: Q5: 82.6%, Q6: 43.6%, R5: 50.7%, R6: 88.9%). The serum of this calf, analyzed one month earlier (prior to the purchase), did not test positive (Q5, −31 dpi, 5.7%). The time points, at which the negative cut-off value was passed by the individual animals, are listed in Table 4.

The ELISA values for the control calf stayed on the same level during the whole experiment (e.g., S2, 28 dpi: −1.1%).

#### 3.3.2. Indirect Immunofluorescence Assay (iIFA)

**Figure 8:**
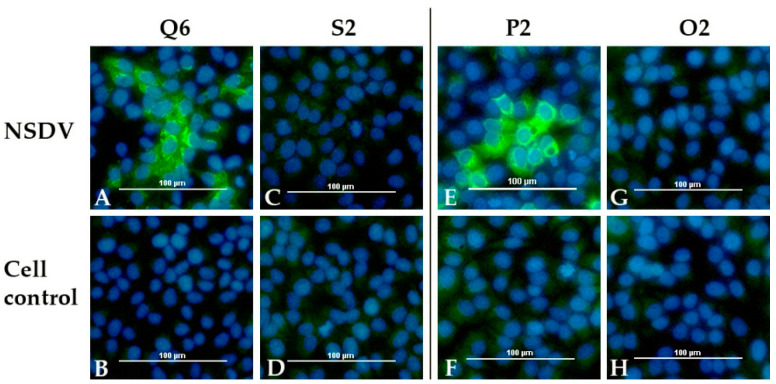
NSDV immunofluorescence assay: Results for one infected calf (Q6) and one infected sheep (P2) are presented together with the results for the mock control animals (calf S2 and sheep O2). Specific staining is shown for both infected animals on cells infected with NSDV (Q6: **A**, P2: **E**). No signal was detected for the control animals (S2: **C**, O2: **G**) and for all animals for non-infected cells (Q6: **B**, S2: **D**, P2: **F**, O2: **H**).

All sera were examined with the indirect immunofluorescence assay (iIFA). A specific staining of NSDV infected Vero E6 cells was seen for all calf sera (Q5, Q6, R5, R6) and for all sheep, which survived until the end of the experiment (N1, N3, N4, P1, P2, P3). Additionally, the serum of sheep N2 was tested as weakly positive at 8 dpi. The sera of the control animals (sheep O1, O2, calf S2) and of all sheep, which were euthanized 7 dpi, did not lead to a specific fluorescence signal. The number of days post infection, when sera were first scored positive, are listed in Table 4. The results for one sheep (P2) and one calf (Q6) are exemplarily depicted, together with the corresponding control animal (O2, S2) (Figure 8).

#### 3.3.3. PRNT

All end-point sera were analyzed with the NSDV PRNT. Sera prior to infection, as well as the sera of the control animals, did not lead to a significant reduction in plaques. Aside from sheep P4, all animals developed neutralizing antibodies. The sheep which were necropsied after one week already revealed PRNT_80_ titers between 1/16 (M2, M4) and 1/128 (N2). The surviving sheep had titers up to 1/4096 (P2). The bovines developed NSDV neutralizing antibodies with titers between 1/32 (Q6) and 1/256 (R5). The immunized animals also developed neutralizing antibodies (NSDV-IMMU-sheep-1: 1/32 and NSDV-IMMU-calf-1: 1/1024). PRNT_80_ titers of all animals are listed in Table 5.

#### 3.3.4. mVNT

**Figure 9:**
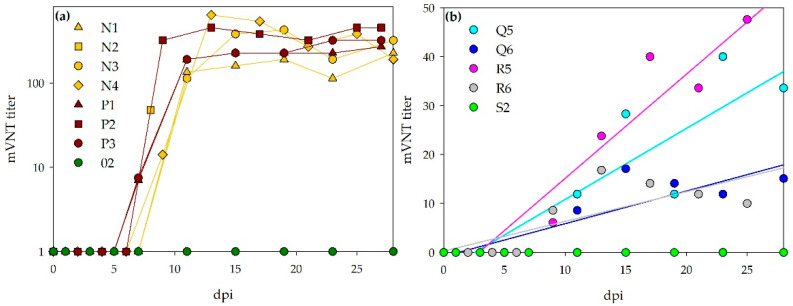
Course of mVNT titers: Panel (**a**) visualizes the rapid increase in mVNT titers for the infected sheep. Panel (**b**) shows the steady increase (linear regression, calculated by SigmaPlot) of mVNT titers for the inoculated calves. Attention has to be paid on the different axis scaling for bovines and ovines.

As a second neutralization test format, a micro-virus neutralization test was performed (Figure 9). Multiple sera can be tested in parallel and, therefore, sera collected at each of the individual time points were analyzed. ND_50_ titers >1/7 were scored positive. Sera prior to infection as well as sera of all control animals during the experiment tested negative.

All sheep that survived until the end of the experiment developed high titers of neutralizing antibodies. The sheep, which were euthanized after 7 dpi were tested negative. However, the serum of sheep N2 at 8 dpi was already tested as weak positive. In general, a rapid increase is seen for all sheep, starting one-week post infection. Thereafter, antibody levels stayed on the same level until the end of the observation period. The calves all developed neutralizing antibodies, but titers were obviously lower than those from the infected sheep. The days post infection, on which sera were first scored positive, are listed in Table 4.

#### 3.3.5. Comparison of Serological Assays

Table 4 shows at which time points post infection sera were first scored positive by the individual assays. There is a nearly perfect concordance between the iIFA and SNT results, the ELISA seems to be slightly less sensitive. All sheep were ELISA-positive at least one sampling point later than with the other assays. The first detection of NSDV antibodies by ELISA was identical with the other assays for two calves but, for the other two calves, the same is true for the sheep sera, with the ELISA being somewhat less sensitive.

## 4. Discussion

In this study, the species-specific diagnosis and pathogeneses of NSDV infections in ruminants were investigated. Utilizing several new diagnostic assays—including RT-qPCR, ELISAs and neutralization assays—the course of NSDV infection was comparatively characterized in sheep and cattle.

The sheep all developed marked clinical signs, including dullness, cachexia, high fever, loss of appetite and diarrhea, comparable to the previously described disease symptoms [6,10,17]. Additionally, gross pathological lesions of sheep killed during the clinical phase of the disease were mainly seen in the gut and gut associated lymphoid tissue. Actually, a more severe course of infection would have been expected for the animals, which received a 100-fold (group M) or 10,000-fold (group P) higher infection dose than the individuals infected with 10 TCID_50_ NSDV only. However, the clinical signs and fever curves did not vary significantly between the three dosage groups, underlining the very high pathogenicity of NSDV in sheep. In fact, all animals of the middle dosage group had to be euthanized at seven dpi, whereas three animals of the high and low dosage group survived until the end of the experiment. No absolute dosage dependent mortality ratios could be calculated, due to the small animal numbers, and the fact that the sheep were indeed euthanized mainly due to animal welfare issues before they succumbed to the disease. Therefore, it cannot be excluded that some of them might have actually survived. In former studies, the development of diarrhea was meant to be a prognostic factor for a fatal disease outcome [10]. This statement could not be confirmed, as six animals with transient diarrhea survived the NSDV challenge and fully recovered afterwards. In contrast, the calves did not show any clinical signs or gross pathology, and no increase in the rectal body temperatures was observed. This is well in line with Montgomery’s observations [10].

The fever period in sheep coincided with the viremic state detected by RT-qPCR analyses. Viral genome equivalents were detected in similar amounts in both serum and whole blood (EDTA) samples, and virus titrations revealed viable virus in several serum samples collected during viremia. In contrast, no viremia was detected in bovines. As the transmission of arboviruses actually requires viremic host species, it could be concluded that bovines do not contribute to the natural infection cycle. However, experimental infections might differ from natural, tick-bite-acquired infections (saliva-assisted transmission) [35]. As previously described, calves did not develop detectable viremia following experimental infections with the closely related DUGV, even if bovines are known to be the main hosts for this arbovirus and DUGV could already be isolated out of cattle blood in Africa [33,35]. Therefore, it cannot be excluded that cattle develop viremia following natural NSDV infections. Future research involving RT-qPCR analyses of sheep and cattle serum samples from endemic regions would further help to address this issue.

As NSDV is an arbovirus, the transmission of the virus is thought to be dependent on vector-competent ticks. Montgomery kept experimentally infected sheep closely together with naïve sheep, but no horizontal transmission was achieved. However, animals could be sufficiently infected with infectious urine (subcutaneous injection) and perorally with feed spiked with viremic blood [10]. Our study revealed that the virus is also shed via the respiratory and alimentary tract, as nasal and rectal swabs of sheep contained high amounts of NSDV RNA. Virus titrations were negative for all collected swab samples, indicating that no infective virus was present, or that the viral loads were below the detection limit. In conclusion, contact transmission between animals is not very likely, but swab sampling (especially nasal swabs) could be an additional non-invasive method for disease diagnosis without the need to handle infectious blood samples. However, human NSDV infections through aerosol transmission cannot be completely ruled out [5] and, therefore, safety precautions should be taken when dealing with this pathogen.

RT-qPCR analyses and virus titrations were also performed to determine the actual virus–organ distribution patterns. The fact that all organs of the sheep euthanized at seven- or eight-days post infection yielded NSDV RNA is probably due to the viremic state at this time. Future pathohistological analyses, including immunohistochemistry (IHC), will reveal, in which organs the virus can indeed be found in the organ parenchyma. However, RT-qPCR analysis still demonstrated a ranking order with highest viral loads in the spleen and liver samples, followed by the intestines and conchae. This is well in line with previous observations, as the spleen and liver had the highest virus contents, when LD_50_ doses in mice were determined for different organs of necropsied sheep [11]. Virus titrations also revealed viable virus particles in different organs, but due to the viremic state of the animals, these results may also not be due to a specific organ distribution pattern. Titrations of the end-point sera of sheep M1 and M3 were already negative at 7 dpi, whereas titrations of several organs were clearly positive then. The results for these sheep can, therefore, give a hint on the actual organ distribution pattern (lung, liver, spleen, ileum, colon, rectum, conchae, conjunctiva, injection site, celiac ganglion, diverse lymph nodes). The six sheep, which survived until the end of the experiment revealed low amounts of NSDV RNA mainly in the lymphatic tissues. For the bovines, only three RT-qPCR positive samples were detected – all of them with low copy numbers in different lymphatic tissues of three calves. As all calves showed seroconversion following NSDV inoculation, these findings are not surprising.

The seroconversion of sheep and bovines was demonstrated by different serological assays, including a newly developed ELISA, based on recombinant N protein, an indirect immunofluorescence assay and two neutralization assays.

The sheep, which survived until the end of the experiment, were tested positive with all of these serological assays. The ELISA seems to be slightly less sensitive than the other assays, as the first detection of antibodies via ELISA was at least one sampling time point later than the first detection by iIFA and SNT. The same observations were already published for an NSDV ELISA, based on whole virus antigen, when comparing the corresponding ELISA, HI and iIFA results [36,37]. Nevertheless, the new N protein-based ELISA is still able to reliably detect anti-NSDV antibodies. The cut-off value for sheep (37.5%) is apparently quite high, because several German sheep already reacted weakly with the ELISA antigen. Future investigations will show whether this protocol is applicable to African/Asian field sera or has to be further adapted. The sheep, which were euthanized at 7 dpi, had no antibodies detectable with ELISA, iIFA or SNT. In contrast, the PRNT could already show low PRNT_80_ titers for these animals and is, therefore, considered to be the most sensitive assay. Sheep N2 (necropsy 8 dpi) did not test positive in the ELISA but did in all other assays. Neutralizing titers were as high as 1/128 in the PRNT and 1/48 in the SNT, but these neutralizing antibodies were nevertheless not capable of preventing fatal disease outcome.

Our data concerning the bovine serology indeed give the first indication that bovines are susceptible hosts and are not resistant to NSDV infections. In general, antibody levels were significantly lower than in sheep, but were comparable to the antibody titers following DUGV infections, as published elsewhere [35]. The actual role of bovines in the natural transmission cycle of NSDV is not well studied or understood yet. Although, in this study, the calves did not develop detectable viremia, virus shedding, or viable virus loads in the analyzed organs, they might contribute to NSDV transmission in nature. Without the requirement of a viremic host species, arboviruses can also be transmitted via non-viremic transmission (NVT), when ticks are co-feeding in close proximity attached to a single host [38]. Moreover, it has been shown that NSDV-infected ticks do not lose their infectivity after feeding on an immune animal [17]. Hence, cattle might not be themselves essential for actual NSDV maintenance in nature but may contribute indirectly when rendering possible the continuous reproduction cycle of NSDV infective ticks.

Considering that NSDV and the human pathogen CCHFV share several features, such as an overlapping host (ruminants) and distribution range (Africa, Asia), as well as genetic and antigenic similarities, there are concerns that NSDV antibodies may interfere in CCHFV assays that are currently in use. CCHFV monitoring programs with ruminants are conducted worldwide to assess the risk of CCHFV infections in humans [39]. Cross-reactions between CCHFV and NSDV have already been observed earlier, even if these viruses actually do not belong to the same serogroups [22,40]. However, serological relationships have not yet been investigated in detail, involving sera from the target species of such monitoring programs (mainly ruminants) and modern diagnostic assays, such as ELISAs or indirect immunofluorescence assays based on CCHFV protein-expressing cells. In fact, the mono-specific antisera obtained during this study are predisposed to address this research issue in subsequent studies.

## 5. Conclusions

In summary, sheep and bovines are both susceptible for Nairobi sheep disease orthonairovirus. Whereas sheep develop severe clinical signs, viremia and shedding of the virus, the course of infection in bovines is only subclinical. Both species show seroconversion, which was demonstrated by different diagnostic assays, which will be useful tools for future monitoring studies. In particular, the newly developed N protein-based ELISAs which do—in contrast to the other assays—not require BSL 3 containment laboratories, will simplify epidemiological investigations. Doubtful results should be confirmed with additional assays, such as iIFA or mVNT. The diagnostic sensitivity, as well as specificity, of the presented assays, will be further evaluated by including panels of field sera. Furthermore, putative cross-reactivities with other orthonairoviruses need to be assessed in future studies.

## Figures and Tables

**Table 1:** Sequences of primers and probes used for the detection of NSDV genome.

**Primer/Probe** **NSDV** **S Segment**	**Sequence 5′ → 3′**	**Localization According to Reference Sequence (GenBank Accession Number: AF504294)**
NSDV-GVF1	TGACCATGCAGAACCAGATYG	47–67
NSDV-GVR1A	GAAACAAGCCTCATGCTAACCT	212–191
NSDV-GV-P1	FAM- CAAGGATGCCATCCTTGCATGGCA -BHQ1	78–101
**Primer/Probe** **Internal** **Extraction** **Control**	**Sequence 5′ → 3′**	**Localization According to Reference Sequence (GenBank Accession Number: MK213795)**
MS2F	CTCTGAGAGCGGCTCTATTGGT	2233–2254
MS2R	GTTCCCTACAACG AGCCTAAATTC	2333–2310
MS2probe	HEX-TCAGACACGCGGTCCGCTATAACGA-BHQ1	2278–2302

**Table 2:** Viral serum titers in sheep during the viremic state.

Sheep	Viral Titers (10^x^ TCID_50_/mL Serum)								
	–Days Post Infection (dpi)–								
	1	2	3	4	5	6	7	8	9
N1	-		2.5		4.0		2.5		
N2				4.25		3.5		-	
N3			2.0		3.75		3.25		
N4				4.0		3.5			-
M1			3.75		3.75		-		
M2				3.5		4.0	1.75		
M3			3.75		4.25		-		
M4		-		4.75		3.75	1.75		
P1			5.0		3.0		-		
P2		2.5		4.5		1.75			-
P3			4.0		3.75		-		
P4		-		3.25		3.5	2.0		

Bright grey: RT-qPCR positive results, dark grey: no survival, -: no CPE.

**Table 3:** Viral titers (10^x^ TCID_50_/100 µL organ homogenate).

Organs	Sheep					
	N2	M1	M2	M3	M4	P4
Lung	-	-	1.0	2.5	-	2.5
Liver	-	-	1.0	1.5	1.5	2.75
Lnn. hepatici	-	2.0	-	2.0	-	-
Spleen	-	2.75	2.5	3.0	0.75	3.0
Jejunum	-	-	-	-	-	1.5
Ileum	-	1.75	-	-	-	-
Colon	0.75	1.0	-	-	-	1.0
Rectum	1.0	2.75	-	-	1.0	-
Lnn. mesenteriales	-	0.75	-	0.75	-	-
Kidney	-	-	-	-	0.75	-
Conchae	1.75	2.25	3.5	4.25	1.25	1.25
Conjunctiva	1.75	1.0	2.0	3.0	-	2.5
Injection site	0.75	1.0	-	1.75	1.0	-
Ln. cervicalis superf.	-	-	-	1.75	-	-
Lnn. iliaci med	-	-	-	1.5	0.75	-
Celiac ganglion	-	1.0	-	1.0	-	-

All organ samples collected during the necropsies of the calves were also analyzed by RT-qPCR. Low equivalents of viral genome copies were detected in lymphatic tissues (R5 Ln. cervicalis cran.: 6 copies/mg; Q5 Lnn. retropharyngeales: 2.5 copies/mg and Q6 Lnn. iliaci mediales: 47.2 copies/mg).

**Table 4:** Comparison of first detection of NSDV antibodies by individual assays.

Challenge Animals		NSDV Diagnostic Assay		
		ELISA (>Cut-Off)	iIFA (≥1/50)	mVNT (≥1/7)
**Sheep**	N1	15 dpi	11 dpi	11 dpi
	N2	Negative	8 dpi	8 dpi
	N3	15 dpi	11 dpi	11 dpi
	N4	13 dpi	9 dpi	9 dpi
	M1	Negative	Negative	Negative
	M2	Negative	Negative	Negative
	M3	Negative	Negative	Negative
	M4	Negative	Negative	Negative
	P1	15 dpi	7 dpi	7 dpi
	P2	13 dpi	9 dpi	9 dpi
	P3	15 dpi	11 dpi	7 dpi
	P4	Negative	Negative	Negative
	O1, O2	Negative	Negative	Negative
**Calves**	Q5	11 dpi	11 dpi	11 dpi
	Q6	23 dpi	11 dpi	11 dpi
	R5	13 dpi	13 dpi	13 dpi
	R6	13 dpi	9 dpi	9 dpi
	S2	Negative	Negative	Negative

**Table 5:** PRNT_80_ titers.

Animal		dpi	PRNT_80_ Titer
**Sheep**	N1	28 dpi	1/2048
	N2	8 dpi	1/128
	N3	28 dpi	1/1024
	N4	28 dpi	1/2048
	M1	7 dpi	1/32
	M2	7 dpi	1/16
	M3	7 dpi	1/32
	M4	7 dpi	1/16
	P1	27 dpi	1/1024
	P2	27 dpi	1/4096
	P3	27 dpi	1/2048
	P4	7 dpi	<1/8
	O1	7 dpi	<1/8
	O2	28 dpi	<1/8
**Calves**	Q5	26 dpi	1/128
	Q6	26 dpi	1/32
	R5	27 dpi	1/256
	R6	27 dpi	1/64
	S2	28 dpi	<1/8
**NSDV-IMMU-sheep-1**		Post-immunization sera after 3 boosts	1/32
**NSDV-IMMU-calf-1**			1/1024

## Data Availability

The data presented in this study are available within this manuscript, Hartlaub et al., Viruses.
